# Tuberculosis of the Right Fifth Metatarsal and Fourth Web Space Without Pulmonary Involvement: A Rare Case

**DOI:** 10.7759/cureus.41160

**Published:** 2023-06-29

**Authors:** Sankalp Yadav, Gautam Rawal, Madhan Jeyaraman

**Affiliations:** 1 Medicine, Shri Madan Lal Khurana Chest Clinic, New Delhi, IND; 2 Respiratory Medical Critical Care, Max Super Speciality Hospital, New Delhi, IND; 3 Orthopaedics, ACS Medical College and Hospital, Dr. MGR Educational and Research Institute, Chennai, IND

**Keywords:** foot bones, osteomyelitis, tuberculosis, fourth web space, fifth metatarsal

## Abstract

Tuberculosis of the bones and joints is an uncommon entity. The bacterial infection of small bones of the foot, like the metatarsals, is extremely rare. Such cases are often detected late, and as a result, there is delayed management. The present case is that of a 12-year-old Indian boy who came with complaints of pain and swelling below his right foot. In the absence of pulmonary involvement, a definite diagnosis of tuberculosis of the fifth metatarsal with fourth web space was established using histopathology, a cartridge-based nucleic acid amplification test, magnetic resonance imaging, and culture of the pus. He was prescribed first-line anti-tubercular treatment for 12 months.

## Introduction

Tuberculosis is a disease known since ancient times that has caused major issues related to public health in high-burden countries [1]. This disease is a result of infection with *Mycobacterium tuberculosis* [1]. Often, it manifests as pulmonary tuberculosis, but extrapulmonary tuberculosis (15-20%) is also common in countries with a high load of this disease [2].

Nearly 1-3% of total extrapulmonary cases are of skeletal tuberculosis [3]. The most commonly involved sites are the spine, hip, knee, and bones of the foot [4]. However, the involvement of small bones in the foot is very rare [5]. Tuberculosis of the foot and ankle accounts for nearly 10% of all osteoarticular tuberculosis [6]. Further, the incidence of tubercular infection of metatarsals is <0.5%, and it is commonly seen in the first and fifth metatarsals [7,8].

The present case is a 12-year-old Indian boy who came with complaints of pain and swelling on the plantar aspect of his right foot. In the absence of pulmonary involvement, a final diagnosis was established using the cartridge-based nucleic acid amplification test, histopathology, magnetic resonance imaging, and culture of the pus. He was initiated on first-line anti-tubercular drugs for 12 months, per the national guidelines.

## Case presentation

A 12-year-old Indian boy from a low-income family was brought in by his parents due to complaints of pain and swelling under the right midfoot for one month. It was difficult for him to bear weight on the affected foot, and this was associated with a visible limp.

He had a history of trauma one and a half months ago when he had a thorn prick. No medical attention was sought at that time, and over-the-counter painkillers like nonsteroidal anti-inflammatory drugs were given to achieve temporary relief of pain. This patient reported to us that this injury burgeoned over time with excruciating pain that was not relieved by nonsteroidal anti-inflammatory drugs. He had no history of any constitutional symptoms of tuberculosis, and there had been no remarkable medical or surgical interventions in the past. Besides, he was fully vaccinated for his age, and there was no history of similar complaints in his family or any contact with tuberculosis.

A general examination revealed an average-build male with a pulse of 71 per minute, a temperature of 98.4 degrees Fahrenheit, a blood pressure of 110/76 mm of Hg, a respiratory rate of 16 per minute, and oxygen saturation (SpO_2_) of 99% in room air.

A local examination disclosed a swollen right fifth toe. The range of movement of the fifth metatarsophalangeal joint, eversion and inversion movements, dorsiflexion, and plantar flexion of the right foot was difficult due to unbearable pain. Further, there were no dilated veins on the skin adjacent to the swelling, but it was erythematous and warm to the touch.

Furthermore, the remaining joints of the right foot were normal, and there were no similar findings on the contralateral foot. And there was no lymphadenopathy, cyanosis, koilonychia, clubbing, pallor, or icterus. A detailed systemic examination was normal.

Based on atypical clinical findings and the initial examinations, a preliminary diagnosis of pyogenic osteomyelitis of the right foot was made with differentials for tubercular osteomyelitis, fungal osteomyelitis, and bone tumour.

**Figure 1:**
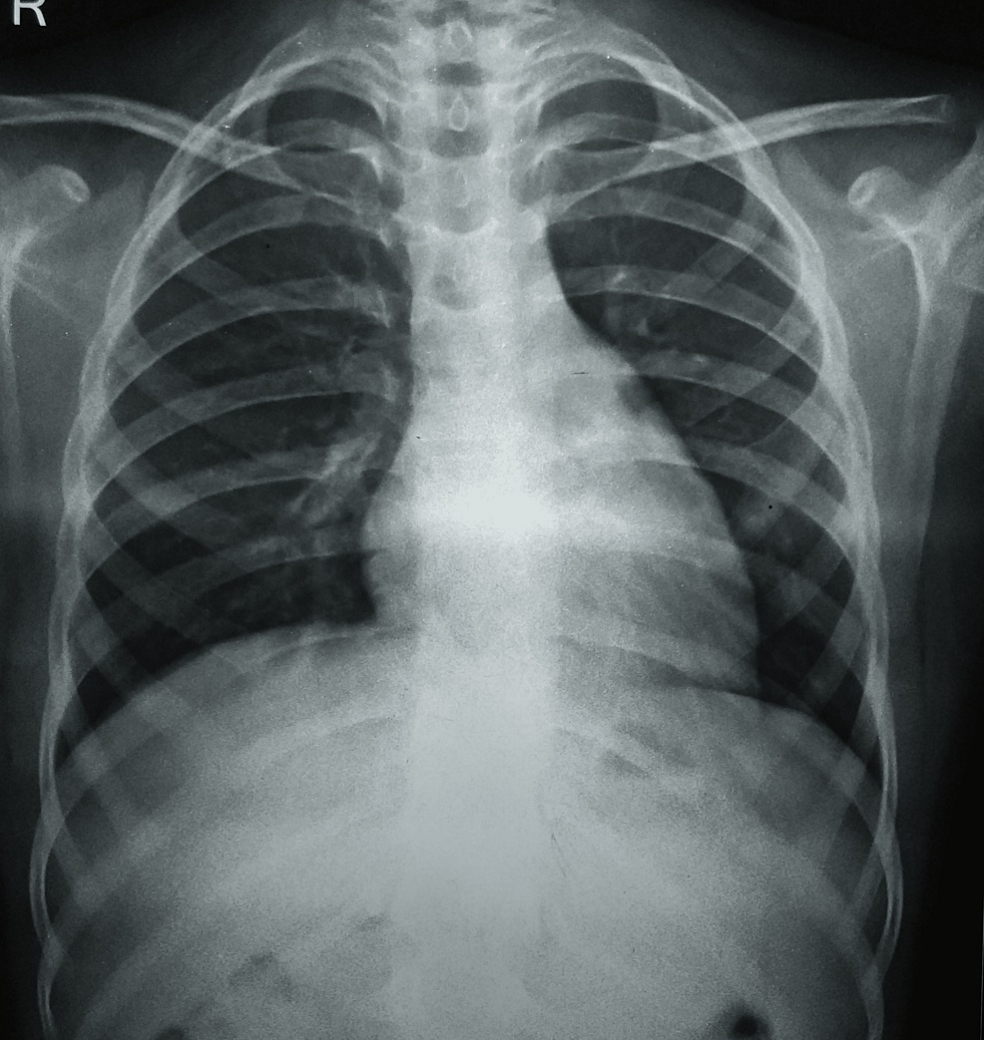
A normal chest radiograph, posteroanterior view

Lab tests were unremarkable except for an upheave of erythrocyte sedimentation rate of 49 mm in the first hour; his induced sputum for acid-fast bacilli and cartridge-based nucleic acid amplification test (CBNAAT) were not suggestive of any infection by *Mycobacterium tuberculosis*. Also, his tuberculin skin test, rheumatoid factor test, and HIV I and II tests were non-reactive. His chest radiograph was not suggestive of any pulmonary infection (Figure 1).

**Figure 2:**
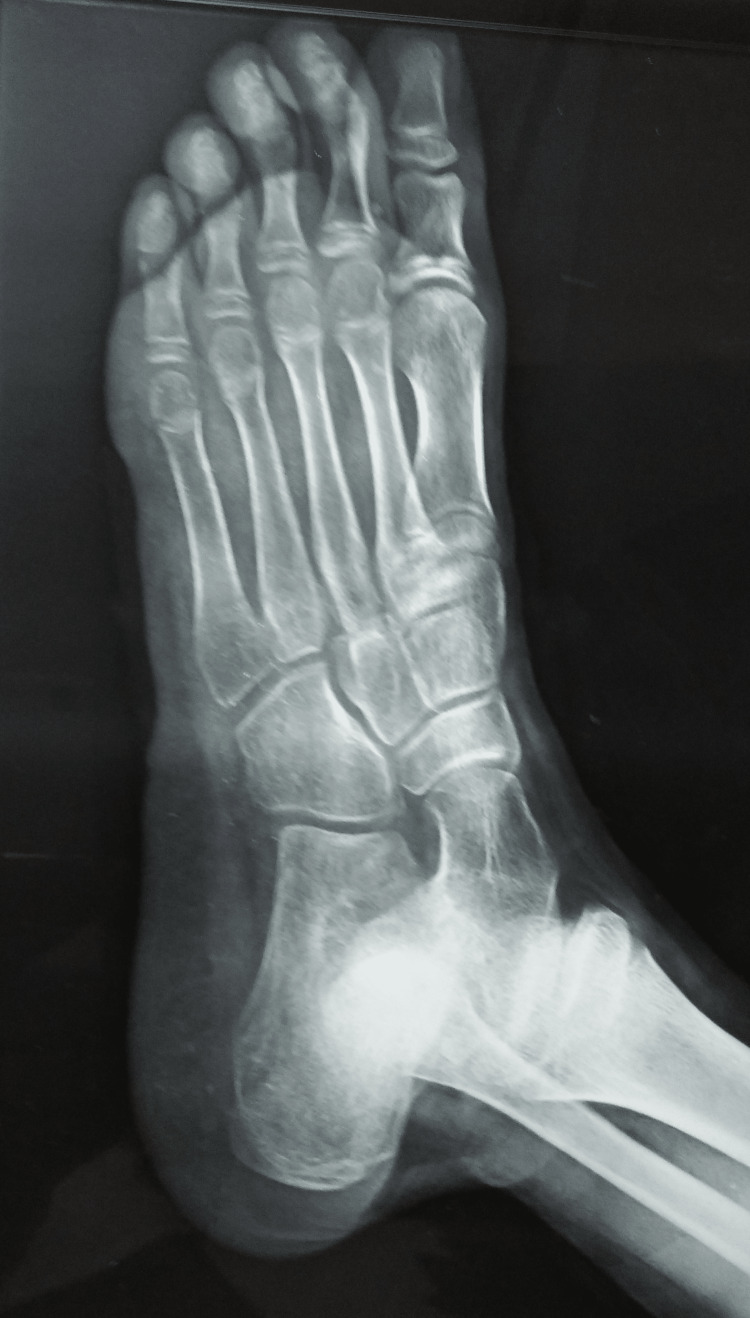
An unremarkable radiograph of the right foot, lateral view

**Figure 3:**
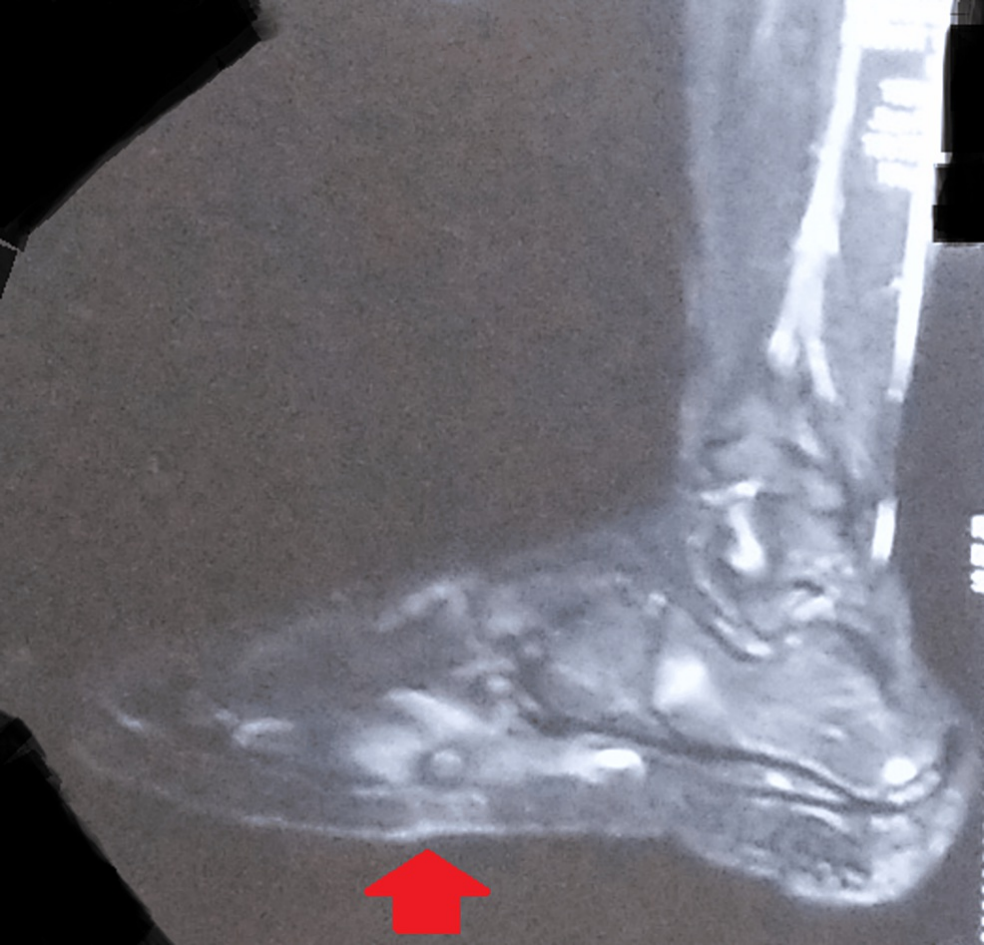
MRI of the right foot showing a hyper-intense sinus tract in the plantar aspect of the midfoot with overlying skin and subcutaneous ulceration with oedema with mild marrow oedema in the fifth metatarsal. MRI: magnetic resonance imaging

Antero-posterior, lateral, and oblique radiographs of the right foot were unremarkable for any lesions (Figure 2). He underwent magnetic resonance imaging of the right foot, which revealed a hyper-intense sinus tract in the plantar aspect of the midfoot with overlying skin and subcutaneous ulceration with oedema with an extension into the muscular plane of the plantar muscles and a blind end. Mild marrow oedema was seen in the fifth metatarsal, but there was no cortical erosion. Mild inflammatory changes were seen in the fourth web space (Figure 3).

He was planned for an open biopsy, and wound debridement was done. This included the excision of sinus tracts and the drainage of about 10 mL of pus. The samples obtained were sent for histopathological and microbiological investigations.

The smear of pus for acid-fast bacilli on Ziehl-Neelsen staining was negative. But histopathology was notable for tuberculosis with caseating necrosis, Langhans giant cells, and epitheloid granulomas with lymphocytes. There was a low detection of *Mycobacterium tuberculosis* with sensitivity to rifampicin on the CBNAAT. The results of the line probe assay (for drug-susceptibility testing for rifampicin and isoniazid resistance) were negative. However, there was *Mycobacterium tuberculosis* grown on liquid culture media with no resistance to the first-line anti-tubercular drugs.

Eventually, a definite diagnosis of tuberculosis of the fifth metatarsal of the right foot with a fourth web space was made. An anti-tuberculous treatment per the national guidelines according to his weight was initiated, initially with four drugs (rifampicin, pyrazinamide, ethambutol, and isoniazid) for 56 doses in the initiation phase, which was followed by a three-drug therapy (rifampicin, ethambutol, and isoniazid) for the next 10 months in the continuation phase. A tablet of pyridoxine (1mg/kg/day) was prescribed for the entire course, and dietary advice for a high-protein diet was also given.

He was followed up for two months, during which he responded positively without any major adverse reaction to anti-tubercular drugs, and after two months, at his parent's request, he was referred to a different district.

## Discussion

Tuberculosis is a substantial contributor to morbidity and mortality in underprivileged countries in Asia, Africa, and Europe [1,5]. Mainly, this affects the lungs, and extrapulmonary infections are reported due to hematogenous spread from the lungs [1,9]. Further, other modes of transmission include a direct extension from adjacent lesions, lymphatic, or either reactivation of dormant tuberculosis bacilli in extrapulmonary organs. Isolated cases at extrapulmonary sites without pulmonary seeding are scarce. Additionally, tubercular infection of the bones of the foot is seldom reported, and commonly involved bones in decreasing frequency of occurrence are the calcaneum, talus, first metatarsal, navicular, and medial and intermediate cuneiforms [10].

Often, the disease has a delayed clinical diagnosis [10,11]. This could well be attributed to subtle clinical features that are often overlapping with other musculoskeletal disorders, ambiguous presentations, paucibacillary characteristics of this bacterial disease, and a lack of management awareness among primary care physicians [11]. Therefore, tuberculosis of the foot bones like the metatarsals is a difficult-to-diagnose disease, and this negatively impacts the management outcomes [10].

Tuberculosis of the metatarsals has a female predilection, and these patients are usually reported in their 20s or 30s [12]. A third of these cases have pulmonary manifestations, which makes it essential to exclude pulmonary involvement in cases with tuberculosis of the metatarsals [9].

Tuberculosis of the metatarsals presents with a confusing picture like an ache at the site of infliction, swelling, and irritation on walking, which often mimics other musculoskeletal disorders and is therefore not disease-specific [4]. Nevertheless, symptoms like a painful swelling affecting a joint or bone with an insidious onset, an unhealed ulcer, and a sinus linked with a discharge should make the treating physicians circumspect about this disease [9].

As seen in this case, the results of radiography techniques are usually vague [4,9]. Common features noted on a radiograph may vary from joint effusion, lytic lesions, and periarticular osteoporosis to bone marrow oedema, tenosynovitis, or soft-tissue collections [4].

The management of tuberculosis of the metatarsals is predominantly non-surgical and involves the use of anti-tubercular medicines [3,5]. But a surgical approach is taken in cases with intractable disease or as a limb salvage method in patients with deformities in hindfoot joints [3].

Dhillon et al. reported metatarsal involvement in only three cases out of 92 patients with foot tuberculosis [13]. Similarly, Mittal et al. reported 18 cases out of 44 patients with metatarsal tuberculosis [14]. In addition to it, a study by Agarwal et al. reported only two cases out of 21 pediatric patients with tuberculosis of the fifth metatarsal [15]. Further, Nayak et al. detected metatarsal involvement in 12 out of 20 cases of ankle and foot tuberculosis [16].

Herein, we present a challenging tuberculosis case where the concomitant involvement of the fifth metatarsal and fourth web space is penned down without pulmonary seeding. In the absence of pulmonary involvement, we attribute the extrapulmonary involvement due to a direct inoculation due to trauma (thorn prick), a hematogenous spread from the lungs, lymphatic spread, or because of reactivation of dormant *Mycobacterium tuberculosis *at the extrapulmonary site. It is emphasized here that the paucity of data related to such presentations should inspire the reporting of similar presentations. However, a single case was the limitation here, and thus large-scale studies from high-burden countries with this rare condition are recommended.

## Conclusions

A case of tuberculosis of the fifth metatarsal with a fourth web space is presented without pulmonary seeding. This case should help fellow clinicians be circumspect about these rare manifestations of a common infection, i.e., tuberculosis beyond the lungs. This case stresses the need for further research related to tuberculosis of the small bones, especially when high-burden countries are aiming for tuberculosis elimination.
